# The Dual Edge of zinc: linking excessive intake to obesity, diabetes, hypertension, and cardiovascular risks

**DOI:** 10.3389/abp.2025.15550

**Published:** 2025-12-11

**Authors:** Tanushree Das, Rhea Ahongshangbam, Romoka Chabungbam, Kshetrimayum Birla Singh

**Affiliations:** 1 Department of Zoology, Sikkim University, Gangtok, Sikkim, India; 2 Department of Zoology, Dhanamanjuri University, Imphal, Manipur, India; 3 Department of Zoology, Manipur University, Canchipur, Manipur, India

**Keywords:** Zn, obesity, diabetes mellitus, hypertension, and cardiovascular diseases

## Abstract

Zinc, an essential trace element, plays a pivotal role in numerous physiological processes, including antioxidant defense, immune regulation, and metabolic homeostasis. However, excessive Zn supplementation disrupts these pathways, contributing to the pathogenesis of chronic conditions such as obesity, diabetes mellitus, hypertension, and cardiovascular diseases. This systematic review explores the dual-edged nature of Zn by examining its molecular impacts, including antioxidant enzyme dysregulation, leptin receptor resistance, and inflammatory marker modulation. While optimal Zn levels confer protective benefits, such as improved insulin sensitivity and reduced oxidative stress, excessive intake triggers systemic inflammation, oxidative damage, and metabolic dysregulation. Contrasting evidence highlights dose-dependent effects and variability based on genetic and environmental factors, underscoring the need for tailored dietary guidelines. Knowledge gaps persist regarding Zn toxicity thresholds, long-term impacts, and interactions with other nutrients. Public health policies must prioritize balanced supplementation strategies to mitigate risks while leveraging Zn’s therapeutic potential in chronic disease prevention. This review emphasizes the importance of precision nutrition and evidence-based approaches to optimize Zn’s benefits while minimizing its adverse effects.

## Introduction

Micronutrients play a crucial role in maintaining overall metabolic health, with deficiencies often linked to the onset of chronic diseases such as obesity, diabetes, and cardiovascular disorders (Romani, 2016). Among these essential micronutrients, Zinc has garnered significant attention due to its wide-ranging physiological functions, from its role in antioxidant defense to its influence on hormonal regulation (Prasad, 2014). Zn is an essential trace element that plays a pivotal role in numerous physiological processes, including enzymatic functions, immune responses, and metabolic regulation. It is widely recognized for its involvement in the structural and functional integrity of antioxidant enzymes, such as superoxide dismutase (SOD), catalase (CAT), and glutathione peroxidase (GPX), as well as its regulatory influence on hormonal systems like leptin signalling (Prasad, 2008; Chausmer, 1998). However, while Zn deficiency has been extensively studied, the potential adverse effects of excessive Zn intake remain underexplored. Recent trends in Zn supplementation in agriculture, animal husbandry, and human diets have raised concerns about its overconsumption.

Zn’s widespread use, particularly in Zn-enriched diets and multivitamin supplements, has led to unintentional exposure to levels exceeding physiological requirements. Such chronic overexposure may disrupt metabolic homeostasis, contributing to oxidative stress, dyslipidemia, and hormonal imbalances (Das et al., 2023). These disruptions are known to underlie the pathophysiology of chronic conditions such as obesity, diabetes mellitus, hypertension, and cardiovascular diseases (CVD). Given the global burden of these non-communicable diseases, it is crucial to investigate the molecular and biochemical effects of excessive Zn intake and its contribution to disease mechanisms.

This review aims to integrate findings from recent studies, including data from our investigation on excessive Zn supplementation in Wistar rats, to elucidate its correlation with the pathophysiological factors resulting in obesity, diabetes, hypertension, and CVD.

## Excessive Zn and its biological effects

Zn is indispensable for numerous biological processes, particularly enzymatic functions, cellular signalling, and gene expression. However, excessive Zn intake can disrupt these processes, leading to detrimental physiological outcomes. Evidence from our research on Wistar rats has demonstrated that chronic Zn overload significantly affects antioxidant enzyme activity, lipid metabolism, and leptin receptor expression, providing a basis for understanding its broader implications.

According to globally accepted dietary guidelines, the Recommended Dietary Allowance (RDA) is 8 mg/day for adult women and 11 mg/day for adult men, with a Tolerable Upper Intake Level (UL) of 40 mg/day. These limits are defined by major regulatory bodies, including the European Food Safety Authority (EFSA, 2014) and the World Health Organization/Food and Agriculture Organization (WHO/FAO, 2004). In experimental research, excessive Zn exposure usually involves doses three to five times higher than normal physiological needs, often amounting to 50–150 mg/kg in rodent models. In human studies, long-term Zn consumption exceeding the UL (≥40 mg/day) is regarded as potentially harmful, particularly due to its association with copper depletion, adverse lipid alterations, and suppressed immune function. These established thresholds provide the basis for interpreting the findings summarized in this review.

Short-term therapeutic doses (50–150 mg/day) are sometimes prescribed for acute infections or deficiency management, but such high doses should not be continued chronically due to the risk of oxidative stress, disruptions in lipid and glucose metabolism, and mineral imbalance. Moreover, emerging clinical observations suggest that continuous high-dose supplementation may contribute to metabolic disturbances, highlighting the importance of cycling zinc supplementation—using it intermittently rather than continuously—to avoid long-term adverse effects. Human-specific dosage ranges, along with temporal patterns of supplementation, therefore play a crucial role in determining whether zinc exerts beneficial or detrimental effects.

### Antioxidant enzyme dysregulation

Zn influences the activity of key antioxidant enzymes that protect against oxidative damage. Excessive Zn levels, however, have been shown to disrupt this balance:

- Cu/Zn Superoxide Dismutase (SOD): Elevated Zn levels resulted in dysregulation of Cu/Zn SOD activity, impairing the neutralization of superoxide radicals. This disruption enhances oxidative stress, a precursor to chronic conditions like cardiovascular diseases (Harris, 2009).
- Glutathione Peroxidase (GPX) and Catalase (CAT): Prolonged Zn supplementation led to reduced GPX and CAT activities in our study, amplifying hydrogen peroxide accumulation. Such oxidative imbalances are associated with endothelial dysfunction and hypertension (Prasad, 2008).

### Leptin and appetite regulation

Leptin is a critical hormone for energy balance, and its signaling pathways are influenced by Zn levels. Our findings revealed upregulation of leptin receptor expression following prolonged Zn exposure (Das et al., 2024). This heightened expression, however, was accompanied by leptin resistance, a condition where leptin’s ability to regulate appetite is impaired (Chausmer, 1998). Elevated serum leptin levels observed in Zn-overloaded rats suggest a disruption in appetite regulation, which contributes to obesity and metabolic syndrome (Friedman and Halaas, 1998).

### Metabolic and biochemical changes

**FIGURE 1:**
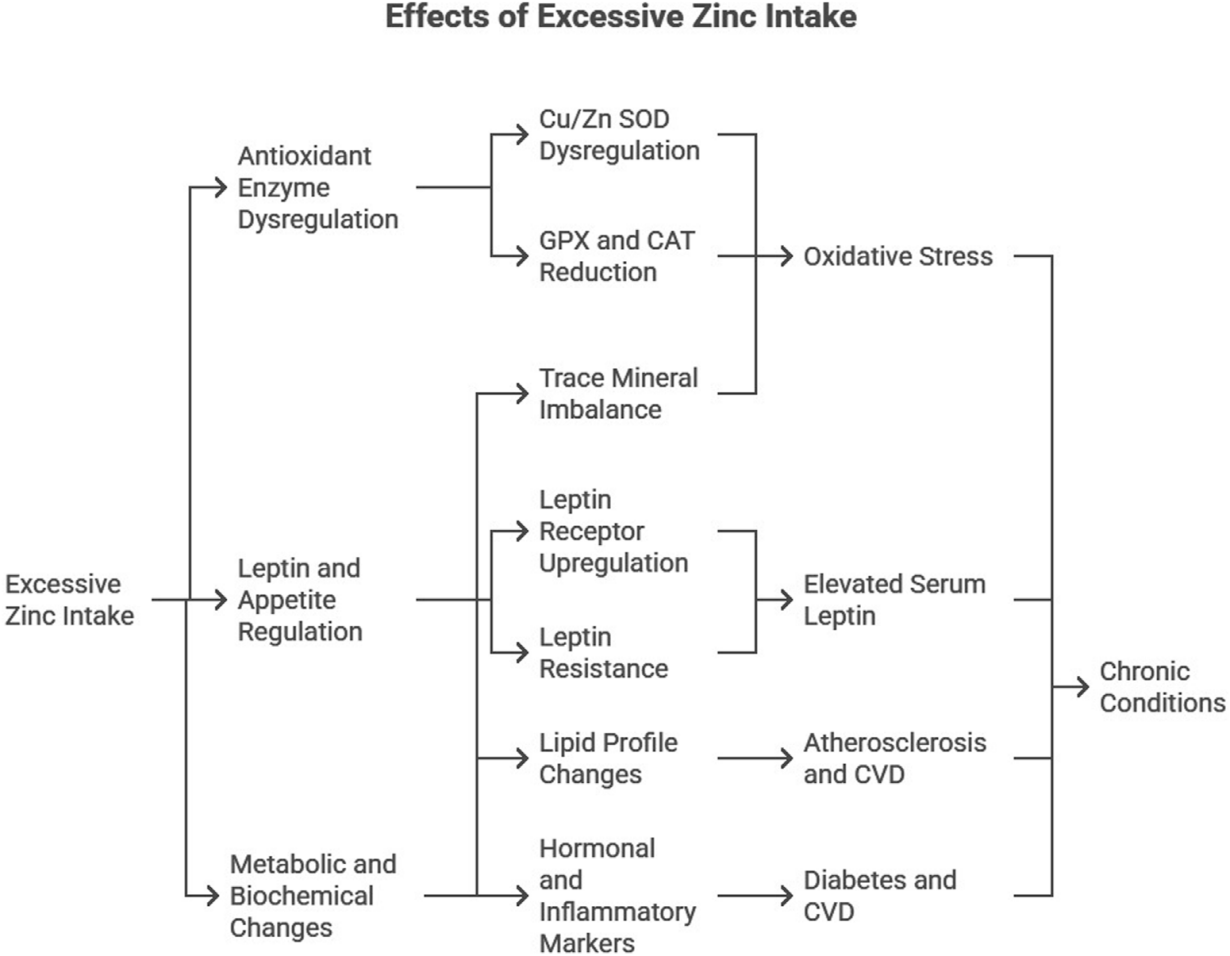
Diagrammatic representation of the effects of excessive Zn intake.

Excessive Zn supplementation induces significant alterations in lipid profiles, hormonal balance, and trace mineral homeostasis (Figure 1):

Lipid Profile: Zn overload increased triglycerides, total cholesterol, and very-low-density lipoprotein (VLDL) levels while reducing high-density lipoprotein (HDL) levels. Such dyslipidemia is a known risk factor for atherosclerosis and cardiovascular diseases (Maret and Sandstead, 2006).

Hormonal and Inflammatory Markers: Elevated serum insulin, HbA1c, and C-reactive protein (CRP) levels were observed, indicating disrupted glucose metabolism and systemic inflammation, key contributors to diabetes and CVD (Hotamisligil, 2006).

Trace Mineral Imbalance: Zn supplementation altered the balance of essential trace elements, such as decreased copper and manganese levels. These competitive interactions exacerbate oxidative stress and metabolic dysfunction (Plum et al., 2010).

These findings highlight the dual role of Zn as both an essential micronutrient and a potential disruptor of metabolic processes when consumed in excess.

## Methods

This review was conducted to summarize current evidence on the effects of excessive Zn intake on obesity, diabetes, hypertension, and cardiovascular diseases. A structured search was performed in PubMed, Scopus, Google Scholar, and Web of Science for studies published between 1990 and 2025. The keywords used in various combinations included “Zn supplementation,” “Zn toxicity,” “obesity,” “diabetes mellitus,” “hypertension,” “cardiovascular diseases,” “oxidative stress,” “leptin resistance,” and “metabolic disorders.”

### Inclusion and exclusion criteria

This review included studies that examined the effects of excessive Zn intake from diet or supplementation and reported outcomes related to obesity, lipid metabolism, insulin resistance, glucose regulation, oxidative stress, inflammation, hypertension, or cardiovascular function. Experimental animal studies, human clinical or observational studies, and mechanistic research published in English were considered. Studies were excluded if they focused solely on Zn deficiency, lacked measurable biochemical or physiological outcomes, or were case reports, conference abstracts, or narrative/commentary pieces without primary data. This selection ensured that only relevant and scientifically robust studies addressing the biological impact of Zn overload were incorporated into the review.

### Data extraction and synthesis

All qualifying studies were screened manually, and relevant data were extracted, including study design, Zn dosage, duration of exposure, biological markers assessed and key findings. Data from our own experimental research on Wistar rats were also incorporated to support the discussion. The extracted information was synthesized narratively, focusing on biochemical pathways, metabolic alterations, and disease mechanisms associated with Zn overload.

## Correlation with pathophysiological factors

Excessive Zn supplementation has been linked to significant biochemical and physiological alterations that contribute to the development of obesity, diabetes mellitus (DM), hypertension (HTN), and cardiovascular diseases (CVD). These chronic diseases often share overlapping pathophysiological pathways, including dyslipidemia, oxidative stress, inflammation, and hormonal dysregulation. These adverse effects are mediated through complex biochemical pathways involving oxidative stress, insulin resistance, dyslipidemia, and renal impairment. This section discusses the molecular and clinical implications of Zn overload in the context of these conditions, integrating findings from the present study and supporting literature, along with recent advancements in the field. Table 1 provides a consolidated overview of the major pathophysiological consequences of excessive Zn supplementation, highlighting its multi-systemic impact on metabolic, endocrine, oxidative, inflammatory, renal, and cardiovascular processes. It summarizes how Zn overload disrupts lipid and glucose homeostasis, impairs antioxidant defenses, induces chronic inflammation, alters mineral balance, and contributes to conditions such as obesity, insulin resistance, diabetes, hypertension, and cardiovascular disease. Each condition is accompanied by the corresponding mechanisms and key literature, offering a clear evidence-based understanding of Zn-induced metabolic and physiological dysregulation.

**TABLE 1:** Effects of excess Zn on biological systems and related pathophysiological mechanisms.

Condition	Effect	Mechanism	References
Obesity	↑ Body fat, visceral adiposity	Impaired glucose clearance, adipocyte hypertrophy	Huang et al. (2017), Chen et al. (1996)
Obesity (dyslipidemia)	↑ TG, ↑ TC, ↑ VLDL-C, ↓ HDL-C	Altered hepatic/lipoprotein lipase activity, lipid metabolism dysregulation	Das et al. (2023), Bhatti et al. (2001), Shabana et al. (2020), Yin et al. (2021), Klop et al. (2013)
Obesity (leptin resistance)	Appetite dysregulation, fat accumulation	Upregulation of leptin receptors, altered IL-6, microbiota dysbiosis	Friedman and Halaas (1998), Huang et al. (2017), Chen et al. (2021), Mohseni et al. (2018)
Obesity (oxidative stress & inflammation)	↑ Oxidative damage, ↑ TNF-α, IL-6, CRP	↓ Cu/Zn SOD & CAT, mitochondrial dysfunction, systemic inflammation	Singh (2012), Furukawa et al. (2004), Xiao et al. (2022), Visser et al. (1999)
Diabetes mellitus	↑ Diabetes and prediabetes risk	Zn–triglyceride–ALT axis; β-cell dysfunction, insulin resistance	Zhang et al. (2021), Yang et al. (2023)
Diabetes (insulin resistance)	↓ Insulin signaling, ↑ hyperglycemia	Impaired GLUT4 translocation, ↓ insulin receptor phosphorylation	Huang et al. (2017), Chen et al. (1996), Wohaieb and Godin (1987), Jayawardena et al. (2012)
Diabetes (oxidative stress & inflammation)	↑ CRP, ↑ lipid peroxidation	↓ GPX and CAT; endothelial dysfunction from hyperglycemia	Koenig et al. (1999), Kahn et al. (2006), Patel and Patel (2015), Mackenzie and Bergdahl (2022)
Hypertension	↑ Mean arterial pressure, ↓ renal function	RAS activation, ↑ kidney AT II levels	Yanagisawa et al. (2014a), Kasai et al. (2012)
Hypertension (oxidative stress)	Endothelial dysfunction, ↑ superoxide	↓ Cu/Zn SOD activity → ↓ NO bioavailability	Harrison and Gongora (2009), Sundaram et al. (2013)
Hypertension (mineral imbalance)	Vascular dysfunction	↓ Copper, magnesium due to Zn overload	Li et al. (2019)
Hypertension (inflammation)	Chronic vascular inflammation	↑ CRP, hs-CRP → elevated BP	Bautista et al. (2001), Sesso et al. (2003), Ebrahimi et al. (2016)
Cardiovascular disease	Atherosclerosis, myocardial injury	↑ Zn:Cu ratio, ZnONPs induce dyslipidemia and vascular damage	Kärberg et al. (2022), Ceballos-Gutierrez et al. (2021)
CVD (oxidative stress)	Cardiac dysfunction, ↑ ROS	↓ CAT activity in myocardium	Baumer et al. (2000), Mackenzie and Bergdahl (2022), Tanski et al. (2023)
CVD (inflammation)	↑ CRP, plaque instability	CRP-mediated endothelial activation; ↑ LDL-C, ↓ HDL-C	Koenig et al. (1999), Hage (2014), Zou (2023), Taneja et al. (2006)
CVD (antioxidant response)	Compensatory enzyme response	↑ Catalase gene expression in failing hearts	Dieterich et al. (2000)

### Obesity

Obesity is characterized by an imbalance between caloric intake and energy expenditure, often exacerbated by hormonal and metabolic dysregulation. Zn’s role in lipid metabolism and appetite regulation implicates it in the pathogenesis of obesity. Dyslipidemia, defined by elevated triglycerides (TG) and low high-density lipoprotein cholesterol (HDL-C), is a hallmark of obesity and a precursor to associated conditions such as metabolic syndrome and CVD.

Excessive Zn intake has been linked to an increase in body fat accumulation and visceral adipose tissue hypertrophy. Studies have shown that chronic high-dose Zn supplementation impairs systemic glucose clearance and increases visceral adipose tissue weight and adipocyte size (Huang et al., 2017). Additionally, Zn supplementation has been found to aggravate body fat accumulation in both genetically obese mice and high-fat diet-induced obese mice, suggesting that Zn may interact with dietary fat consumption to exacerbate obesity (Chen et al., 1996).

#### Dyslipidemia and Zn overload

Our study demonstrated that excessive Zn intake leads to significant dyslipidemia, with increased TG, total cholesterol, and very-low-density lipoprotein cholesterol (VLDL-C) levels, alongside decreased HDL-C levels (Das et al., 2023). These findings align with reports by Bhatti et al. (2001) and Shabana et al. (2020), who observed similar lipid abnormalities in obese individuals. The Framingham Study further emphasizes that low HDL-C levels are a critical risk factor for coronary heart disease (Rabkin et al., 1981). Recent studies have reinforced these observations. For instance, Yin et al. (2021) conducted lipidomic profiling of obese adolescents and found elevated TG levels coupled with dysregulated phospholipid metabolism, directly linking obesity to cardiovascular risks. Moreover, Foster et al. (2010) highlighted that excessive Zn influences the activity of hepatic enzymes, promoting lipid synthesis and reducing lipid clearance.

**FIGURE 2:**
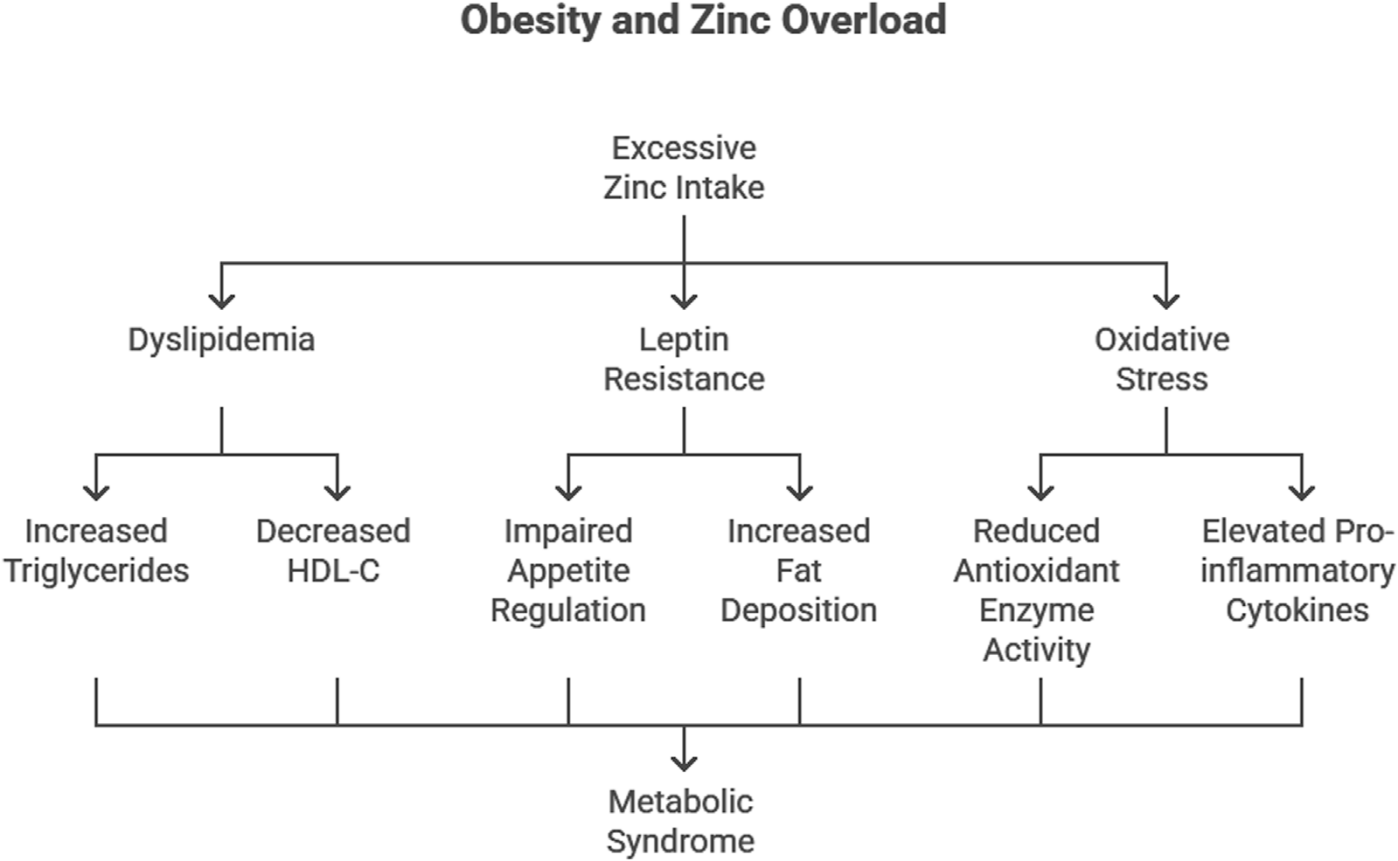
Effect of excess Zn supplementation on body weight and visceral adiposity in experimental models.

Zn’s interaction with lipid metabolism is mediated through its impact on key enzymes. Excess Zn alters the activity of enzymes involved in lipid synthesis and clearance, including hepatic lipase and lipoprotein lipase. This disruption promotes TG accumulation and decreases HDL-C synthesis, contributing to obesity-related dyslipidemia (Klop et al., 2013) (Figure 2).

#### Leptin resistance

Leptin, a hormone secreted by adipocytes, plays a pivotal role in appetite regulation and energy homeostasis. Excessive Zn supplementation was found to upregulate leptin receptor expression in Wistar rats, a compensatory response to leptin resistance. Elevated leptin levels, coupled with resistance to its effects, lead to impaired appetite regulation and increased fat deposition (Friedman and Halaas, 1998) (Figure 2).

The mechanisms by which Zn contributes to obesity may involve the modulation of leptin and interleukin-6 (IL-6) expression in visceral adipose tissue. Elevated leptin levels have been observed in Zn-supplemented mice, which may contribute to fat accumulation and insulin resistance (Huang et al., 2017). Furthermore, Zn-induced dysregulation of gut microbiota has been implicated in metabolic disturbances, including obesity and insulin resistance (Chen et al., 2021).

Recent findings by Mohseni et al. (2018) confirmed that excessive Zn intake alters the expression of Mn-SOD and CAT genes, which are involved in redox signaling. This dysregulation contributes to leptin resistance by impairing hypothalamic signaling pathways.

#### Oxidative stress and inflammation

Oxidative stress is a key driver of obesity-related complications. Previous study revealed that excessive Zn intake disrupts the antioxidant defense system, particularly Cu/Zn superoxide dismutase (SOD) and catalase (CAT), increasing oxidative damage (Singh, 2012). Furukawa et al. (2004) similarly reported reduced antioxidant enzyme activity in adipose tissue of obese mice, highlighting the link between oxidative stress and obesity. Elevated C-reactive protein (CRP), a marker of systemic inflammation, further underscores the inflammatory milieu in obesity (Visser et al., 1999) (Figure 2).

More recent studies, such as that by Xiao et al. (2022), have elucidated how chronic oxidative stress induced by Zn overload contributes to mitochondrial dysfunction in adipocytes, amplifying systemic inflammation. Elevated levels of pro-inflammatory cytokines, including tumor necrosis factor-α (TNF-α) and interleukin-6 (IL-6), were observed in Zn-overloaded states, further exacerbating obesity-related complications.

### Diabetes mellitus

The relationship between Zn and diabetes is complex, with both deficiency and excess Zn linked to an increased risk of diabetes. Type 2 diabetes mellitus (T2DM) is a multifactorial disorder characterized by insulin resistance, hyperglycemia, and dyslipidemia. Zn’s dual role in insulin signaling and oxidative stress regulation underscores its involvement in diabetes pathogenesis.

Serum Zn concentrations have been positively associated with prediabetes and diabetes, with the highest quartile of serum Zn showing a 3.24-fold increased risk of prediabetes and a 1.64-fold increased risk of diabetes (Zhang et al., 2021). A nested case-control study further revealed that higher serum Zn levels are associated with a 51% increased risk of diabetes, with triglycerides and alanine aminotransferase (ALT) acting as mediators (Yang et al., 2023).

#### Insulin resistance and Zn

**FIGURE 3:**
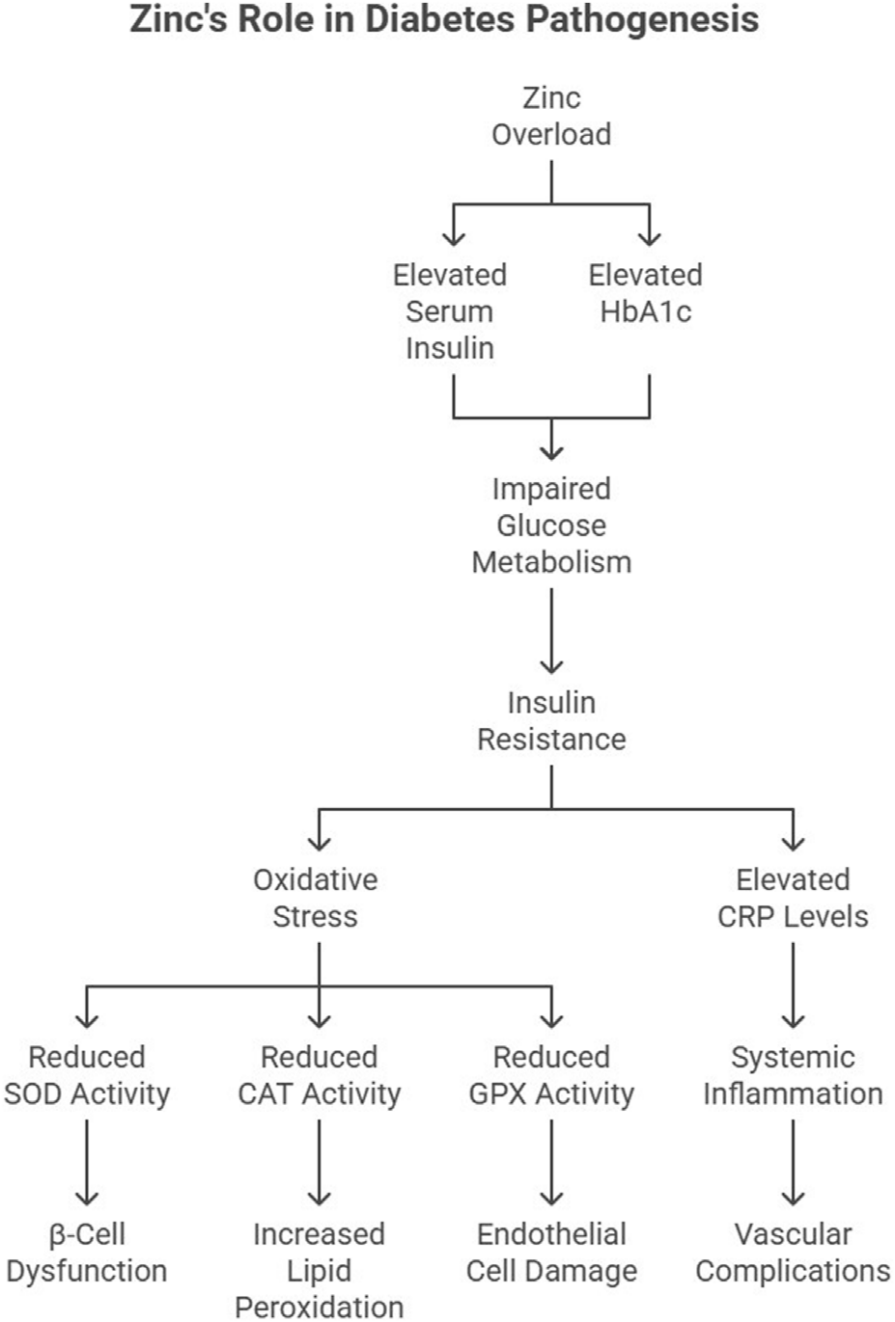
Role of excess Zn in the pathogenesis of diabetes.

The mechanisms by which excessive Zn contributes to diabetes may involve insulin resistance and pancreatic beta-cell dysfunction. Zn supplementation has been shown to impair insulin signaling and glucose metabolism, leading to hyperglycemia and insulin resistance (Huang et al., 2017; Chen et al., 1996). Our findings indicated elevated serum insulin and HbA1c levels in Zn-overloaded rats, suggesting impaired glucose metabolism (Figure 3). Insulin resistance, a hallmark of T2DM, is exacerbated by oxidative stress and inflammation. DeFronzo and Ferrannini (1991) proposed that insulin resistance is a unifying factor linking obesity, hypertension, and diabetes.

Zn’s effect on insulin signaling is complex. While optimal Zn levels enhance insulin secretion and receptor sensitivity, excessive Zn disrupts these processes by increasing oxidative stress in pancreatic β-cells. Studies by Wohaieb and Godin (1987) demonstrated reduced SOD activity in diabetic rat pancreas, correlating with β-cell dysfunction.

Recent research by Jayawardena et al. (2012) identified that excessive Zn supplementation can reduce insulin receptor phosphorylation, impairing glucose uptake. Additionally, hyperZnemia-induced oxidative stress affects glucose transporter (GLUT4) translocation in skeletal muscle, exacerbating hyperglycemia.

#### Oxidative stress in diabetes

Excessive Zn intake may induce oxidative stress and inflammation, which are key contributors to the development and progression of diabetes (Mackenzie and Bergdahl, 2022). The antioxidant system plays a crucial role in mitigating hyperglycemia-induced oxidative damage. However, excessive Zn intake compromises antioxidant enzyme activities, including GPX and CAT, exacerbating oxidative stress. Asayama et al. (1989) reported similar findings, where diminished CAT activity in diabetic tissues correlated with increased lipid peroxidation.

Elevated CRP levels in Zn-overloaded rats further link inflammation to T2DM. CRP is not only a marker of systemic inflammation but also a predictor of diabetes onset (Koenig et al., 1999). Elevated CRP levels in metabolic syndrome patients underscore the inflammatory nature of T2DM (Kahn et al., 2006) (Figure 3). More recently, Patel and Patel (2015) demonstrated that hyperglycemia-induced oxidative stress downregulates CAT and GPX gene expression in endothelial cells, accelerating vascular complications in diabetic patients (Figure 3).

### Hypertension

**FIGURE 4:**
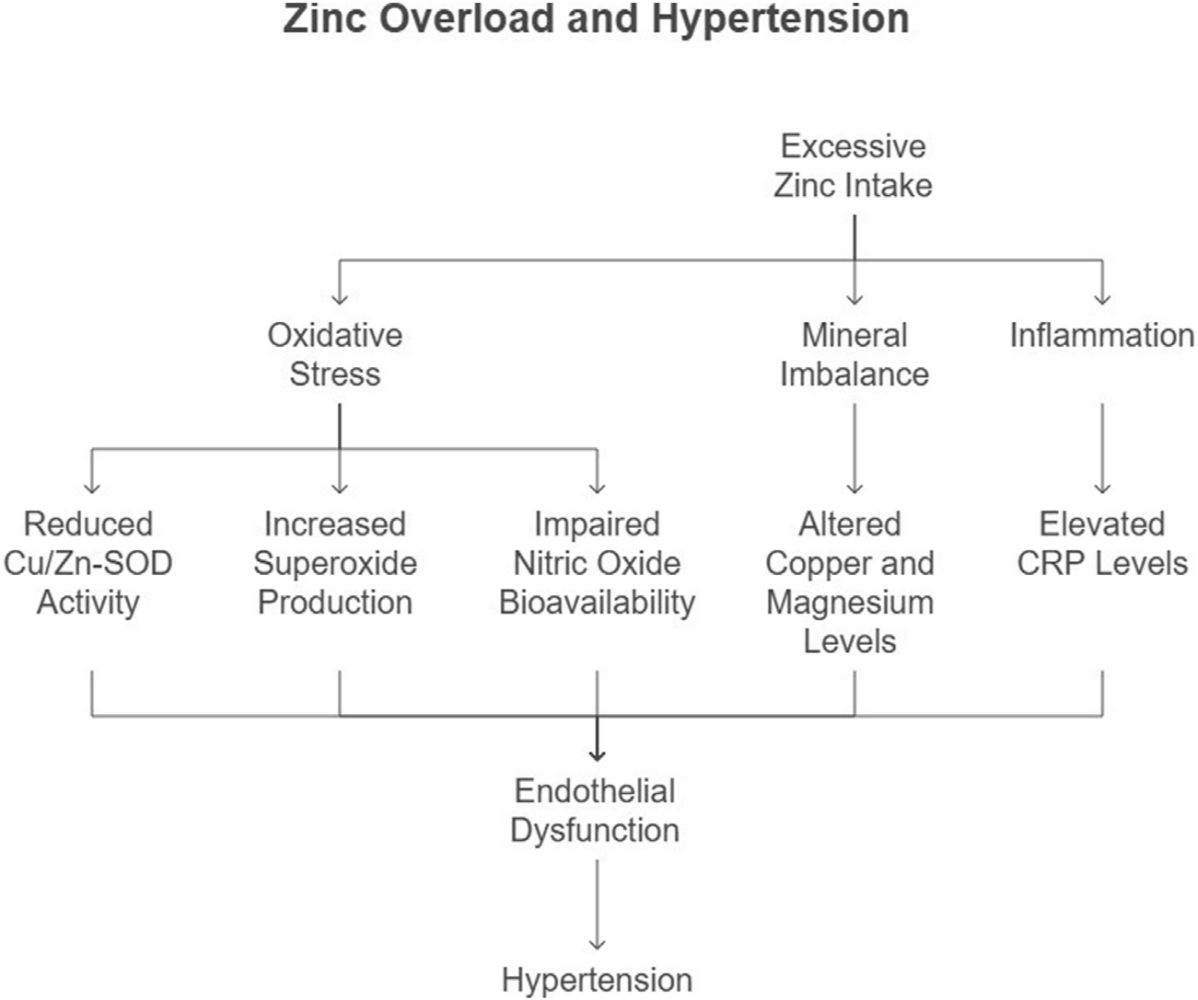
Flow chart illustrating the mechanistic pathway linking excessive Zn consumption to the development of hypertension.

Hypertension, a leading risk factor for CVD, is characterized by increased vascular resistance and endothelial dysfunction. Zn’s role in blood pressure regulation is mediated through its effects on oxidative stress, mineral balance, and vascular function (Figure 4). Excessive Zn intake has been linked to an increase in systemic blood pressure and a reduction in renal blood flow. Studies have shown that Zn-excess diets lead to a dose-dependent increase in mean arterial pressure (MAP) and a decrease in inulin clearance, suggesting impaired renal function (Yanagisawa et al., 2014a) (Kasai et al., 2012). The mechanisms underlying Zn-induced hypertension may involve the renin-angiotensin system (RAS), as kidney angiotensin II (AT II) levels have been found to increase significantly with excessive Zn intake (Kasai et al., 2012).

#### Oxidative stress and endothelial dysfunction

Additionally, Zn-induced oxidative stress may play a role in hypertension. Excessive Zn intake has been shown to reduce the activity of copper/Zn-superoxide dismutase (Cu/Zn-SOD), an endogenous antioxidant enzyme, leading to increased superoxide radical production and subsequent oxidative stress (Yanagisawa et al., 2014b). This oxidative stress may impair nitric oxide (NO) bioavailability, leading to vasoconstriction and increased blood pressure (Yanagisawa et al., 2014a).

Our study demonstrated that Zn overload disrupts the Cu/Zn SOD pathway, increasing superoxide production and reducing nitric oxide (NO) bioavailability. This oxidative imbalance contributes to endothelial dysfunction, a precursor to hypertension (Harrison and Gongora, 2009). Yanagisawa et al. (2014b) further highlighted the role of Zn-induced oxidative stress in elevating systemic blood pressure (Figure 4). Recent evidence from Sundaram et al. (2013) suggests that altered renal CAT and GPX activity precedes the onset of hypertension in Zn-overloaded models, further implicating oxidative stress in its pathogenesis.

#### Mineral imbalance and hypertension

Excessive Zn intake alters the balance of essential trace elements, including copper and magnesium, which are critical for vascular function (Figure 4). A meta-analysis by Li et al. (2019) revealed that hypertensive patients exhibit lower serum Zn levels, emphasizing the complex interplay between Zn and mineral homeostasis. Disruption of this balance by Zn overload exacerbates hypertension risk.

#### Inflammation and hypertension

Chronic inflammation, marked by elevated CRP levels, is a key contributor to hypertension. Our findings of increased CRP in Zn-overloaded rats align with studies by Bautista et al. (2001) and Sesso et al. (2003), who demonstrated a direct correlation between CRP and hypertension risk (Figure 4). These findings underscore the inflammatory basis of Zn-related hypertension.

Recent findings by Ebrahimi et al. (2016) further corroborate the association between elevated hs-CRP levels and hypertension, emphasizing the role of systemic inflammation in blood pressure regulation.

### Cardiovascular diseases

Cardiovascular diseases encompass a range of conditions, including atherosclerosis, myocardial infarction, and heart failure, often rooted in dyslipidemia, oxidative stress, and inflammation. Zn’s influence on these pathways highlights its dual role as both a protective and disruptive factor.

#### Dyslipidemia and atherosclerosis

A high dietary Zn-to-copper (Zn:Cu) ratio has been associated with an increased risk of atherosclerosis, as it may lead to an imbalance in lipid metabolism and oxidative stress (Kärberg et al., 2022). Additionally, Zn oxide nanoparticles (ZnONPs) have been shown to induce dyslipidemia and atherosclerotic lesions, leading to changes in vascular contractility and cannabinoid receptor expression (Ceballos-Gutierrez et al., 2021). The lipid abnormalities observed in our study—elevated TG, total cholesterol, and VLDL-C levels—are established risk factors for atherosclerosis. High TG and low HDL-C levels, as reported by Shabana et al. (2020), accelerate plaque formation and vascular occlusion. The Framingham Study further emphasizes that lipid abnormalities are central to CVD risk (Gordon et al., 1977).

Recent studies by Ozyildirim and Baltaci (2023) highlighted that Zn deficiency, rather than excess, may contribute to CVD in specific populations, underscoring the complexity of Zn’s role. However, Zn overload remains detrimental by promoting oxidative stress and inflammation.

#### Oxidative stress and myocardial dysfunction

Oxidative stress is a key driver of myocardial dysfunction. Excessive Zn intake reduces antioxidant enzyme activity, increasing oxidative damage in cardiac tissues. Baumer et al. (2000) reported decreased CAT activity in dilated cardiomyopathy patients, highlighting the role of oxidative stress in heart failure. Elevated oxidative markers in Zn-overloaded rats reinforce these findings. Furthermore, Zn-induced oxidative stress and inflammation may contribute to endothelial dysfunction and the progression of atherosclerosis (Mackenzie and Bergdahl, 2022; Tanski et al., 2023).

#### Inflammation and CVD

Inflammation plays a central role in CVD pathogenesis. Elevated CRP levels, observed in our study, are predictive of future coronary events (Koenig et al., 1999). CRP not only reflects systemic inflammation but also directly contributes to atherogenesis by promoting endothelial activation and plaque instability (Hage, 2014). The mechanisms by which excessive Zn contributes to cardiovascular risks may involve the modulation of lipid metabolism and vascular function. Excessive Zn intake has been shown to increase low-density lipoprotein cholesterol (LDL-C), triglycerides, and total cholesterol, while decreasing high-density lipoprotein cholesterol (HDL-C) (Zou, 2023; Taneja et al., 2006).

Furthermore, Dieterich et al. (2000) demonstrated that upregulation of antioxidative enzyme gene expression, such as catalase, occurs as a compensatory mechanism in failing hearts, reflecting the intricate interplay between oxidative stress and myocardial injury.

## Mechanistic insights

Excessive Zn intake profoundly impacts molecular pathways, disrupting homeostasis and contributing to the pathogenesis of chronic diseases (Figure 1). This section provides a detailed discussion of key mechanisms, including antioxidant enzyme dysregulation, leptin receptor overexpression and resistance, and the impact on inflammatory markers such as C-reactive protein (CRP).

### Antioxidant enzyme dysregulation

Zn plays a crucial role in the antioxidant defense system by supporting the activity of enzymes such as Cu/Zn superoxide dismutase (SOD). However, excessive Zn disrupts this balance by:

- Inhibiting Cu/Zn SOD Activity: Zn overload competes with other essential trace elements, such as copper and manganese, impairing the function of Cu/Zn SOD. This inhibition leads to the accumulation of superoxide radicals, increasing oxidative stress.
- Reducing Catalase (CAT) and Glutathione Peroxidase (GPX) Activities: Elevated Zn levels diminish the expression and activity of CAT and GPX, impairing hydrogen peroxide detoxification. This disruption exacerbates oxidative damage in cellular membranes, proteins, and DNA, as observed in Zn-overloaded Wistar rats and corroborated by Furukawa et al. (2004).
- Inducing Lipid Peroxidation: By weakening antioxidant defenses, excessive Zn promotes lipid peroxidation, further contributing to endothelial dysfunction and atherosclerosis.

### Leptin receptor overexpression and resistance

**FIGURE 5:**
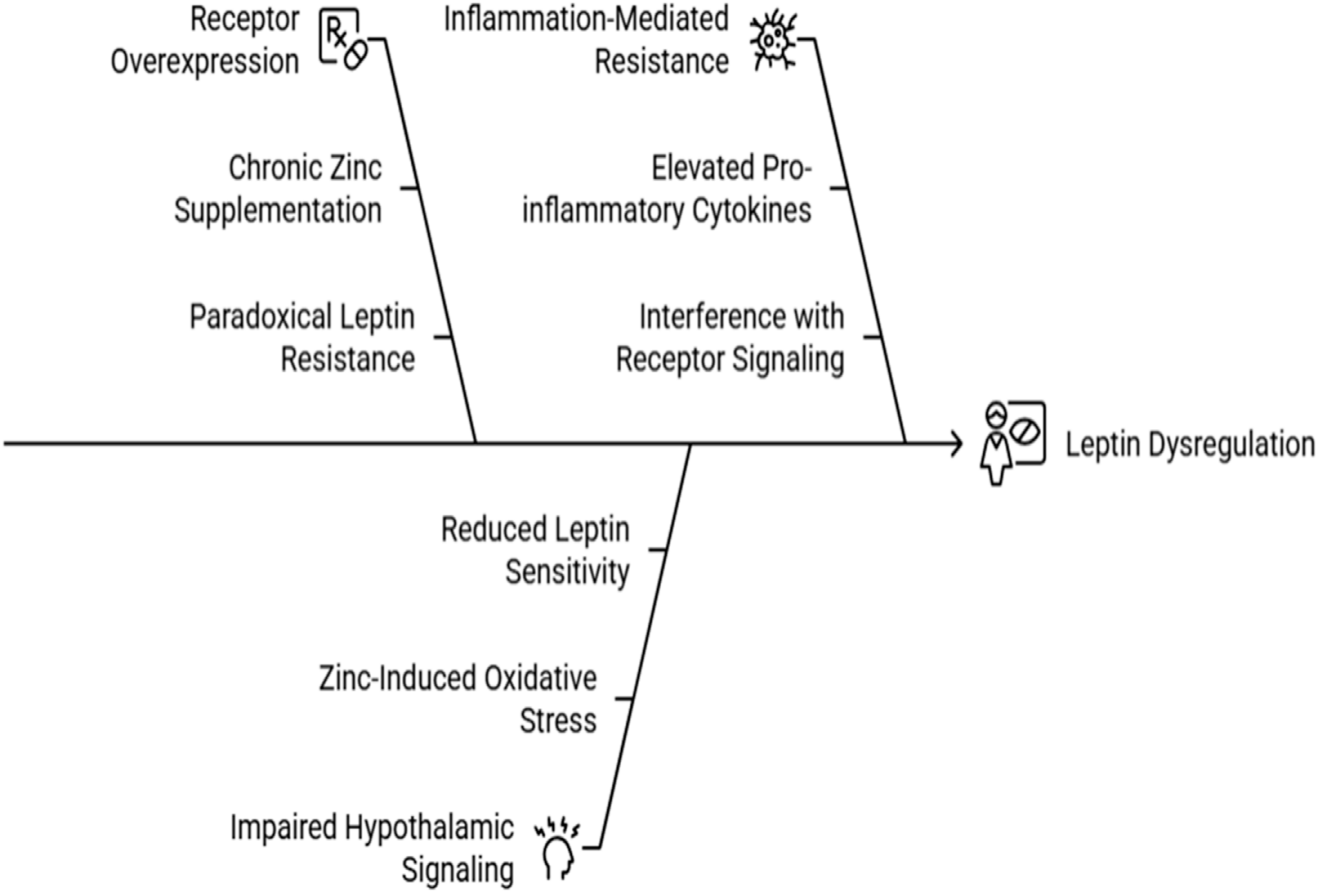
Causes of Leptin dysregulation under Zn Overload.

Leptin, a hormone regulating energy balance, becomes dysregulated under Zn overload. Key mechanisms include:

- Receptor Overexpression: Chronic Zn supplementation upregulates leptin receptor expression, leading to a paradoxical state where elevated leptin levels fail to suppress appetite, causing leptin resistance (Figure 5). Studies in rodent models highlight this compensatory overexpression as a driver of hyperphagia and obesity (Friedman and Halaas, 1998).
- Impaired Hypothalamic Signaling: Zn-induced oxidative stress disrupts redox signaling in the hypothalamus, a critical site for leptin action. This impairment reduces leptin sensitivity, further contributing to metabolic dysregulation (Mohseni et al., 2018) (Figure 5).
- Inflammation-Mediated Resistance: Pro-inflammatory cytokines such as tumor necrosis factor-α (TNF-α) and interleukin-6 (IL-6), elevated in Zn-overloaded states, exacerbate leptin resistance by interfering with receptor signaling pathways (Figure 5).

### Impact on inflammatory markers like CRP

C-reactive protein (CRP) is a sensitive biomarker of systemic inflammation and cardiovascular risk. Zn overload influences CRP levels through:

- Induction of Systemic Inflammation: Excess Zn amplifies oxidative stress, triggering the release of pro-inflammatory cytokines. These cytokines stimulate hepatic CRP production, elevating systemic inflammation.
- Promotion of Atherogenesis: Elevated CRP levels enhance endothelial activation, promoting the recruitment of monocytes and the formation of atherosclerotic plaques. Koenig et al. (1999) demonstrated that high CRP levels predict future coronary events, linking inflammation to cardiovascular disease (CVD).
- Feedback on Metabolic Dysfunction: Elevated CRP levels exacerbate insulin resistance and lipid abnormalities, creating a vicious cycle of metabolic and inflammatory dysregulation.

### Contrasting evidence and knowledge gaps

While the detrimental effects of excessive Zn supplementation are well-documented, conflicting evidence highlights its protective role at optimal levels. This section explores these discrepancies and identifies areas requiring further exploration.

## Discussion

This review synthesizes current evidence on the dual role of Zn in human metabolic and cardiovascular health, highlighting both its essential physiological functions and its potential toxicity when consumed in excess. While optimal Zn intake supports antioxidant defense, immune regulation, glucose metabolism, and lipid homeostasis, chronic overconsumption disrupts these same systems, contributing to obesity, insulin resistance, hypertension, and cardiovascular diseases. The findings reiterate the importance of dose-dependent effects of Zn, yet they also expose significant gaps in the translation of mechanistic findings into clinically meaningful human outcomes.

Zn’s protective role at optimal levels is multifaceted and involves significant contributions to antioxidant defense, immune function, and insulin sensitivity. For instance, Zn activates enzymes like Cu/Zn superoxide dismutase (SOD), which play a critical role in reducing oxidative stress and protecting against cellular damage (Figure 1). Research by Maret and Sandstead (2006) underscores Zn’s ability to mitigate oxidative injury, particularly in chronic conditions such as diabetes and cardiovascular diseases (CVD). Additionally, Zn’s involvement in immune regulation is evident through its impact on cytokine production and T-cell activity, as highlighted in clinical trials where Zn supplementation reduced the duration and severity of infections. Zn also enhances insulin receptor signaling, improving glucose uptake and reducing the risk of type 2 diabetes (Chausmer, 1998). Despite these benefits, evidence from Zn supplementation studies reveals conflicting findings. While animal models provide valuable mechanistic insight into oxidative stress, leptin signaling, and mineral imbalance, their physiological responses do not fully replicate human metabolic processes. Rodent studies often use Zn doses several-fold higher than typical human exposures and exhibit faster metabolic turnover, making direct extrapolation difficult. Human studies show similar trends—such as altered lipid metabolism and impaired copper homeostasis—but the magnitude of effect is smaller. Therefore, findings from animal studies must be interpreted with caution, particularly regarding dosage thresholds and long-term toxicity in humans.

Across included studies, excessive Zn intake consistently induced oxidative stress through dysregulation of antioxidant enzymes, particularly reduced Cu/Zn SOD, CAT, and GPX activities (Furukawa et al., 2004; Singh, 2012). The resulting accumulation of reactive oxygen species (ROS) contributes to endothelial dysfunction, metabolic inflexibility, and increased cardiovascular risk (Harrison and Gongora, 2009; Mackenzie and Bergdahl, 2022). Zn overload also interferes with glucose–insulin homeostasis. Elevated Zn levels impair insulin receptor phosphorylation and GLUT4 translocation (Jayawardena et al., 2012), contributing to insulin resistance and hyperglycemia. Human studies corroborate these associations, showing increased risk of prediabetes and diabetes with higher circulating Zn (Zhang et al., 2021; Yang et al., 2023). In terms of lipid metabolism, excessive Zn promotes dyslipidemia characterized by elevated triglycerides, VLDL-C, and total cholesterol alongside reduced HDL-C (Das et al., 2023; Shabana et al., 2020). Such alterations are mechanistically linked to hepatic enzyme dysregulation affecting lipid synthesis and clearance (Klop et al., 2013), thereby augmenting atherosclerotic risk (Gordon et al., 1977). Inflammatory biomarkers, particularly CRP, were consistently elevated in Zn-overloaded states, aligning with epidemiological evidence that CRP predicts future coronary events and hypertension (Koenig et al., 1999). Finally, Zn-induced mineral imbalance—especially reductions in copper and magnesium—further amplifies oxidative stress and vascular dysfunction (Plum et al., 2010; Li et al., 2019). These combined effects suggest a multilevel disruption of metabolic and cardiovascular physiology under Zn excess.

Despite strong mechanistic evidence for Zn toxicity, findings across the literature remain context-specific. Moderate Zn supplementation improves insulin sensitivity, immune function, and antioxidant capacity (Prasad, 2014; Maret and Sandstead, 2006). Beneficial effects are particularly noted in populations with baseline Zn deficiency, malabsorption, or chronic inflammation. Conversely, adverse effects predominantly occur in Zn-sufficient individuals with chronic high intake, high dietary Zn-to-copper ratios (Kärberg et al., 2022), or intake from multiple fortified sources. These discrepancies reflect differences in baseline nutritional status, genetic and environmental modifiers, Zn formulation (e.g., ZnSO_4_ vs. ZnO nanoparticles), duration of exposure, and study design. They also highlight the need for individualized and population-specific guidelines rather than universal supplementation strategies.

While a substantial portion of the mechanistic evidence in this review is derived from animal models, particularly rodent studies, it is important to acknowledge the inherent limitations of extrapolating these findings directly to humans. Rats are valuable models for studying metabolic regulation, oxidative stress pathways, and trace-element interactions due to their physiological similarity and controllable experimental conditions. However, species-specific differences in zinc absorption, hepatic metabolism, endocrine regulation, and inflammatory responses may influence the magnitude or direction of zinc’s effects. Therefore, while animal studies provide critical insights into underlying biochemical pathways, these findings should be interpreted cautiously when considering human physiology. Inclusion of human observational and interventional studies helps strengthen the translational relevance, but further controlled clinical trials are needed to clarify zinc’s dose-dependent effects in humans. Also a key concern in the reviewed literature is the heavy reliance on animal models, many of which employ supraphysiological doses unlikely to be encountered in typical human diets. Their metabolic rate, gastrointestinal absorption, and trace mineral kinetics differ substantially from humans, limiting external validity. Furthermore, long-term human trials assessing chronic Zn toxicity are scarce, often small in sample size, heterogenous in dosage, and short in duration.

The limited number of rigorous observational or randomized controlled trials (RCTs) evaluating chronic, moderate Zn excess constrains the strength of causal inference in humans. Additionally, publication bias toward positive or mechanistic outcomes may overrepresent harmful or beneficial effects in isolation. Recent epidemiological and clinical studies have provided additional support for the role of zinc dysregulation in metabolic disorders. For example, higher serum zinc concentrations have been associated with increased risks of prediabetes and diabetes in large human cohorts (Zhang et al., 2021; Yang et al., 2023), suggesting that zinc overload may be clinically relevant even in free-living populations. Similarly, population-based studies evaluating dietary and supplemental zinc intake have shown that excessive zinc consumption, particularly from long-term supplementation, may contribute to altered lipid profiles, impaired glucose regulation, and increased inflammatory burden. These findings underscore the need to contextualize animal-derived mechanistic data within human physiological variability and highlight the importance of further human trials to clarify optimal intake thresholds.

Because the evidence in this review was derived from systematically searched studies, the strength of conclusions is influenced by variability in study design, zinc doses, and population characteristics. Animal studies were abundant, whereas human trials were fewer and often short-term, limiting direct translation. Nonetheless, systematic inclusion of all eligible studies minimized selection bias and allowed for a balanced assessment of both beneficial and adverse effects. Future research should prioritize (i) long-term human cohort studies evaluating chronic moderate Zn exposure, (ii) dose–response analyses integrating Zn–copper interactions, (iii) mechanistic studies exploring the interplay between Zn and gut microbiota, and (iv) refined models that better account for human physiological Zn kinetics. Nutrigenomic research may also clarify inter-individual variability in Zn metabolism and disease susceptibility.

Given increasing use of fortified foods, dietary supplements, and agricultural Zn enrichment, the potential for unintentional chronic Zn excess warrants public health attention. Supplementation strategies should prioritize at-risk groups while avoiding excessive or indiscriminate use in the general population. Incorporating Zn intake monitoring and regulation of fortification programs could mitigate long-term risks. Precision nutrition approaches, leveraging genetic and metabolic profiling, may guide safer and more effective Zn use.

## Conclusion

This systematic review underscores the dual-edged nature of Zn supplementation in human health. While Zn is essential for numerous physiological processes, including antioxidant defense, immune regulation, and metabolic homeostasis, excessive intake disrupts these functions and contributes to the pathogenesis of obesity, diabetes mellitus, hypertension, and cardiovascular diseases. The mechanistic insights presented reveal how Zn overload dysregulates antioxidant enzymes, exacerbates inflammation, and induces hormonal resistance, creating a cascade of adverse health outcomes. Conflicting evidence regarding Zn’s protective and detrimental effects highlights the importance of dose-dependent and context-specific considerations. Optimal Zn levels confer significant health benefits, such as enhanced antioxidant capacity and improved insulin sensitivity, while excessive intake poses risks, including oxidative stress, dyslipidemia, and systemic inflammation. These findings emphasize the need for individualized approaches to Zn supplementation, taking into account genetic, dietary, and environmental factors. Addressing the knowledge gaps in Zn research requires longitudinal studies to evaluate its long-term effects, as well as mechanistic investigations to clarify its interactions with other trace elements. Public health policies must balance the risks and benefits of Zn supplementation by establishing clear dietary guidelines, promoting targeted interventions for at-risk populations, and regulating Zn fortification in foods. Therapeutic applications of Zn, particularly in combination with other antioxidants, hold promise for mitigating chronic disease burden and improving health outcomes globally.

In conclusion, zinc’s role in health and disease is a dynamic interplay of its essentiality and potential toxicity. Excessive zinc intake has been shown to contribute to obesity, diabetes, hypertension, and cardiovascular risks through multiple biochemical mechanisms, including oxidative stress, insulin resistance, dyslipidemia, and renal dysfunction. While moderate zinc intake is essential for maintaining metabolic and cardiovascular health, excessive zinc supplementation may lead to adverse effects. Therefore, caution is advised when considering zinc supplementation, and further research is needed to determine the optimal dosage and duration of zinc intake for different populations. A nuanced understanding of this trace element is crucial for harnessing its benefits while minimizing its risks, paving the way for precision nutrition and evidence-based public health strategies.
